# Circular RNA Hsa_circRNA_101996 promotes the development of Gastric Cancer via Upregulating Matrix Metalloproteinases-2/Matrix Metalloproteinases-9 through MicroRNA-143/Ten-eleven translocation-2 Pathway

**DOI:** 10.7150/jca.62121

**Published:** 2021-09-21

**Authors:** Feng Huang, Jiajia Jiang, Yongliang Yao, Shiyue Hu, He Wang, Ma Zhu, Liya Yu, Qingqian Liu, Haoyuan Jia, Wenrong Xu

**Affiliations:** 1 Jiangsu Key Laboratory of Medical Science and Laboratory Medicine, School of Medicine, Jiangsu University, 301 Xuefu Road, Zhenjiang, Jiangsu 212013, China.; 2 Department of Clinical Laboratory, The First People's Hospital of Kunshan Affiliated with Jiangsu University, Kunshan, 215300, China.; 3 Aoyang Institute of Cancer, Jiangsu University, 279 Jingang Road, Suzhou, 215600, Jiangsu, China.; 4 Cancer Research Institute of Wuhan, The Central Hospital of Wuhan, Tongji Medical College, Huazhong University of Science and Technology, Wuhan 430014, China.; 5 Department of Clinical Laboratory, Wuxi People's Hospital Affiliated to Nanjing Medical University, Wuxi, China.

**Keywords:** gastric cancer, hsa_circRNA_101996, microRNA143, ten-eleven translocation-2, matrix metalloproteinases

## Abstract

**Background:** The long-term survival rate of gastric cancer (GC) patients at advanced stages remains low worldwide. Circular RNAs (circRNAs) a newly studied type of non-coding RNA that play an important role in the pathogenesis and diagnosis of various diseases. In this research, we aimed to explore the functions of hsa_circRNA_101996 in GC cells and an animal model of GC.

**Methods:** The expression of hsa_circRNA_101996, microRNA (miR)-143, and ten-eleven translocation (TET)-2 in GC tissues, the adjacent tissues, and cell lines were determined by quantitative reverse transcription-polymerase chain reaction (qRT-PCR). Transwell assays were used to analyze the knockdown effects of hsa_circRNA_101996, miR-143, and overexpression of TET2 on cell proliferation, migration, and invasion abilities. Western blotting was used to analyze the expression of matrix metalloproteinases (MMP)2/MMP9. Binding interactions between, hsa_circRNA_101996 and miR-143 and between, miR-143 and TET2 were detected by Dual-luciferase reporter assays. Levels of protein expression were analyzed by Western blotting. Tumor models were established by subcutaneous injection of tumor cells in Bl6/Rag2/GammaC double knockout mice.

**Results:** The result showed that hsa_circRNA_101996 expression was significantly upregulated in GC tissues compared to that in the adjacent tissues, and its level in cancer tissue was correlated with tumor size, lymphatic metastasis, and distant metastasis. Compared with the low hsa_circRNA_101996 expression group, the three-year survival rate of patients in the high hsa_circRNA_101996 expression group was significantly lower. The knockdown of hsa_circRNA_101996 dramatically suppressed the cell migration, invasion, and proliferation of GC cells by sponging to absorb miR-143 and elevated the expression of TET2. *In vivo* studies showed that the knockdown of hsa_circRNA_101996 delayed tumor growth. Furthermore, we revealed that TET2 regulates MMP2/MMP9 expression through the DNA demethylation pathway.

**Conclusion:** Our findings indicate that hsa_circRNA_101996 promotes GC development by upregulating MMP2/MMP9 through miR-143/TET2 pathway, which may provide a novel target for GC.

## Introduction

Gastric cancer (GC) is presently the fifth most common cancer worldwide and the third-highest cause of cancer-related deaths globally 1. The documented survival of GC is greater than 90% for patients at the early stage, but the survival decreases to less than 20% when patients are diagnosed in the advanced stage, which is directly associated with tumor metastasis 2.

Although current treatment options for GC cancer such as surgery, radiotherapy, chemotherapy, targeted therapy, and immunotherapy are constantly updated, the therapeutic effect is limited 3. As a result, the long-term survival rate of GC patients at advanced stages remains low worldwide 4. Therefore, further reveal the molecular mechanisms underlying the occurrence and development of GC cancer is urgent.

CircRNAs are newly discovered endogenous non-coding RNAs which play an important role in pathological and physiological processes of cell proliferation, cell cycle, metastasis, migration, invasion, and so on 5. There is increasing evidence shows that circRNAs play important regulators in the process of cancer development by sponging microRNAs to regulate mRNA translation 6. However, in GC, the role and mechanisms of hsa_circRNA_101996 are not clear.

MicroRNA (miR) is a 21 to 23 nucleotide long RNA molecule that is widely found in eukaryotes and regulates the expression of other genes. Increasing studies have reported that miRs play an important role in regulating gene expression, cell cycle, and the metabolic process by specific binding with target mRNA that furthermore inhibits post-transcriptional gene expression. For example, circDLGAP4 regulated lung cancer cell migration and invasion by sponging miR-143 to modulate cyclin-dependent kinase 1 expression 7. Interestingly, miR-143 was the target of hsa_circRNA_101996 in our study through an online bio prediction site. However, the specific role and molecule mechanism of hsa_circRNA_101996 regulating miR-143 in gastric cells remains unknown.

Ten-eleven translocation (TET)-2 is one member of the TET family of proteins, which also includes TET1 and TET3. TET2 is a crucial driver of cell fate outcomes in a myriad of biological processes through catalyzing the demethylation of 5-methylcytosine on DNA, affecting transcriptional regulation 8. Cancer cell invasion is the fundamental of tumor metastasis. During the metastasis process, cancer cells should first degrade the extracellular matrix with the help of matrix metalloproteinases (MMPs) 9. Among the MMPs family, the MMP2 and MMP9 are of special importance because of their ability to degrade collagen and proteoglycan 10. Previous studies have shown that the DNA methylation status of MMP2 and MMP9 genes are aberrant in tumor tissues 10. DNA methylation, the most common epigenetic modification, could interact with other epigenetic modifications such as histone modification, to regulate the functioning of the genome by changing chromatin architecture 11. For mammals, the DNA methylation generally occurs in cytosine within CpG dinucleotides which are concentrated in large clusters called CpG islands. In normal cells, DNA methylation assures the proper regulation of gene transcription to maintain stable gene silencing, however, in cancer cells, the aberrant DNA methylation within the promoter regions may lead to the inactivation of certain tumor-suppressor genes or induce genomic instability and contributes to cell transformation 12. To date, the role of TET2 in GC has never been studied.

In this study, we evaluated the expression status of hsa_circRNA_101996 in GC tissues and cell lines, and explore the functions and molecular mechanisms of CircRNA-101996/miR-143/TET2 in GC progressions, so as to find the potential therapeutic targets and prognostic biomarkers. Our data suggest hsa_circRNA_101996 transcription is enhanced in GC tissues and cells. hsa_circRNA_101996 inhibited cell proliferation and migration of GC cells. Mechanistically, hsa_circRNA_101996 sponging miR-143 upregulated TET2 expression, thereby promoting the DNA methylation of MMP2/MMP9 in GC cells. Therefore, CircRNA-101996 was able to promote malignant progressions *in vivo* and *in vitro* during the development of GC.

## Materials and Methods

### Ethical approval

The ethical approval, the review of informed consent, the implementation of experimental protocols, and subsequent research of this study have been approved by the Clinical Medical Research Ethics Committee (Approval number: L-D18007). Informed consent was obtained from each patient included in the study, and the study conforms according to The Code of Ethics of the World Medical Association (Declaration of Helsinki), printed in the British Medical Journal (18 July 1964).

### Human tissue and plasma collection

The inclusion criteria for GC patients are: (1) Histopathologically confirmed GC; (2) Patients who completed follow-up (three years); (3) Patient who has provided informed consent; (4) No serious heart, lung, liver, kidney, and other complications; (5) No history of mental illness. The exclusion criteria for GC patients are: (1) Follow-up time is less than three years; (2) History of cancer or radiotherapy and chemotherapy treatment; (3) Patients or their families are reluctant to participate in this study. A total of seventy-nine patients with GC were included in this study, and three cases were excluded after screening by inclusion and exclusion criteria. In the end, a total of seventy-six paired GC and adjacent tissues were obtained from GC patients. Among them, 37 were males and 39 were females. The average age is 53.3±5.9 years. Besides, the plasma was collected from 76 patients with GC before and after surgery, and at the same time, plasma from 76 healthy subjects was collected.

### Patient follow-up

Seventy-six patients were followed up for three years (2016-2019), and the time of death of the patients was recorded.

### Xenograft model

Six-week male Bl6/Rag2/GammaC double knockout mice were purchased (Cyagen, China) and adaptively fed for one week. Unlike NOD-SCID mice, these mice lack T-, B- and NK-cells but show no spontaneous tumor formation and have a normal hematopoietic function. Mice were housed in a facility with a 12 h light/dark cycle maintained at 25±0.5 °C and 50% to 60% humidity.

The xenograft GC model was established as described before 13. Briefly, mice were anesthetized a ~0.5 cm incision was made in the mid-abdomen to expose the stomach. Tumor cells were prepared as a single cell suspension. For each mouse, 1×10^6^ cells in 50 µl matrigel were injected into the serious side of the stomach. Then the stomach was returned to the abdominal cavity and the incision was closed layer by layer using absorbable sutures. After the surgery, mice were continued fed for 6 weeks. The tumor size was measured using a vernier caliper.

For tumor metastasis experiments, approximately 2×10^6^ cells of the transfected cells were injected intraperitoneally into 6-8 week old nude mice. Three weeks later, the mice were sacrificed.

### Cell culture, transfections, and transductions

Human GC cell lines including KATO III (CLS Cat# 300381/p610_KATO-III, RRID: CVCL_0371), MGC-803 (RRID:CVCL_UI43), SGC-7901 (RRID: CVCL_RK36), SNU-520 ((KCLB Cat# 00520, RRID: CVCL_5072)), GTL16 (RRID:CVCL_7668), NCI-N87 (RRID:CVCL_WH01), and MKN-28 (JCRB Cat# NIHS0325, RRID:CVCL_1416) were cultured in Dulbeccos Modified Eagles Medium (DMEM, Thermo Fisher Scientific, USA) supplemented with 10% fetal bovine serum (FBS) (Clark, USA). Human normal gastric epithelium cell line GES-1 (RRID: CVCL_EQ22) was also cultured in DMEM containing 10% FBS. Cells were all placed in an incubator at 37 °C with 5% CO_2_.

Short hairpin RNA for hsa_circRNA_101996 (shCircR_101996), small interfering RNA for miR-143 (simiR-143), and TET2 (siTET2) were purchased (Hippobio, China) and were used to knock down the target transcription. Mimic for miR-143 was also purchased (Hippobio, China) and was used to overexpress miR-143. The transfection was conducted using Lipofectamine 3000® Transfection Reagent (Invitrogen, USA) according to the manufacturers' instructions. The primer sequences used for simiR-143 and siTET2 were as follows: 5′-AGCATGGTCCGCGTATCGCGTdTdT-3′ (forward) and 5′-ACGCAATTGUUCGGAGAAdTdT-3′ (reverse) for simiR-143; 5'-GTGCGGCACTTGCCGGACAATCdTdT-3' (forward) and 5'-CCCGGTGTCACTGCGCACGdTdT-3' (reverse) for siTET2.

pPLK-CircR_101996, pPLK-miR-143, and pPLK-TET2 were constructed by Public Protein/Plasmid Library (Jiangsu, China) to permanently knockdown hsa_circRNA_101996, miR-143, and TET2 respectively. pLV-CircR_101996 and pLV-miR-143 were used to permanently overexpress hsa_circRNA_101996 and miR-143 respectively. The empty vector was used as the control (NC). All these vectors were packaged with lentivirus. Viral supernatants were used to transduce cells. Stable transductions were selected by growth in media containing Ampicillin.

### Subcellular localization of CircR_101996

Subcellular localization of CircR_101996 was identified with fluorescence *in situ* hybridization (FISH) method using a Ribo™ FISH Probe Mix (Green) (RiboBio) according to the manufacture's protocol. Then, the nuclear and cytoplasmic RNA was separated to further confirm the distribution of CircR_101996 in cells.

### Western blot

The proteins of TET2, MMP2, MMP9, and Tubulin were tested by Western blot. The rabbit anti-TET2 (1:2000), MMP2 (1:1000), MMP9 (1:1000), and Tubulin (1:3000) antibodies were added overnight at 4 °C. The secondary antibody was then added and incubated at room temperature for 0.5 h the next day. Immunoreactive bands were visualized with an ECL kit (Promoter, Wuhan, China) and pictured by GeneGnome5 Chemiluminescence Series Image Capture (Syngene, Frederick, MD, USA) according to manufacturers' instructions. Blot densitometric analysis was done by ImageJ software (NIH, USA).

### Chromatin immunoprecipitation (ChIP)

ChIP was established as described before 14. Briefly, cells were cross-linked using 4% methanol for 10 minutes and sheared by sonication to produce DNA fragments of appropriate length (~500bp). 1% of the chromatin fragments were separated as the input. The precipitation product was identified by Western blot. The primer sequences used for ChIP were as follows: 5′-AACTTTCTCACTAGGTGGACdTdT-3′ (forward) and 5′-GCCCATACCTGACGCTGTGAGAdTdT-3′ (reverse) for MMP2-CpG1; 5′-ACTGGCTTTTTGGACACCCAGTCCdTdT-3′ (forward) and 5′-ATCGCGCCGCCGCCACCTGTTGAdTdT-3′ (reverse) for MMP2-CpG2; 5'-GGCATCAACGGCATCGAdTdT-3' (forward) and 5'-CTCGTTCGGTCACCGCCAGTAdTdT-3' (reverse) for MMP9; and 5'-CACGTTCGGCTCGGCGCAdTdT-3' (forward) and 5'-AACGGCTCACCAGTTGCCTdTdT-3' (reverse) for TET2.

### RNA extraction and RT-qPCR analysis

Total RNA of cell lines, tissues, and plasma were separated by TRIzol (15575835, Invitrogen, USA), and then cDNA was further synthesized by PrimeScriptTM RT reagent Kit (RR042A, TaKaRa, Japan). Pre-synthesized gene primers (Sangon, China), Roche SYBR Green Master (05729017211), and DEPC water were added to the cDNA and mixed together and tested in the detection instrument (thermal cycler T100, Bio-Rad, USA), according to the following settings: pre-denaturation at 95 °C for 10 minutes (min), denaturation at 95 °C for 15s, annealing at 58 °C for 1min, for a total of 40 cycles. Glyceraldehyde-3-phosphate dehydrogenase (GAPDH) and U6 were used as the housekeeping genes. The primer sequences used for qRT-PCR were as follows: 5′-CGCCACGGTTTCAGCTGGCGCAC-3′ (forward) and 5′-CAGCTCGTATGACGATCATACG-3′ (reverse) for hsa_circRNA_101996; 5'-GTTCGACGGATCGTTCGT-3' (forward) and 5'-TGTCCGCAGGTTGCACGA-3' (reverse) for MMP2; 5'-CTCGCCTAGGGCAGGCCT-3' (forward) and 5'-AGTGGCTTCACATTAGGCTT-3' (reverse) for MMP9; 5′-GCGCCTTCCTGCAGGAGCCT-3′ (forward) and 5′-TGAGCTTTGATTTCGGAAGCA-3′ (reverse) for miR-143; 5'-GCTTCTCCGCCATGCAAGC-3' (forward) and 5'-AGTTTCGCTGTGTCAAGTGAA-3' (reverse) for TET2; 5'-CGATCGCTTGCACAGCTAGC-3' (forward) and 5'-AGTACGCAGTACGAATAGTGC-3' (reverse) for GAPDH; 5'-GGTCGACGCCCTATGGCACGACTT-3' (forward) and 5'-AACGCTTCACGAATTTGCGT-3' (reverse) for U6. The amplification efficiencies of hsa_circRNA_101996, MMP2, MMP9, miR-143, TET2, GAPDH, and U6 were 97.2%, 94.6%, 103.7%, 101.3%, 104.7%, 96.0%, and 98.1%, respectively. The relative level was calculated by 2^-Δ CT^ method.

### Methylation-specific polymerase chain reaction (MSP)

MSP was performed as described before 15. Genomic DNA was extracted from GC cells using conventional method. The EZ DNA Methylation-GoldTM kit (ZYMO, Beijing, China) was used to convert DNA. TaqMan Universal Master Mix II (Thermo Fisher Scientific, CA, USA) was used to perform all PCR reactions.

### Dual-luciferase reporter gene assay

hsa_circRNA_101996 and TET2 3'UTR containing miR-143 binding sites were constructed into pGL3 vectors (Shanghai Yihui Biological Technology Co., Ltd., Shanghai, China) to form wide type (WT)-hsa_circRNA_101996 and TET2 3'UTR-WT. Mutant type (Mu)-hsa_circRNA_101996 and TET2 3'UTR-Mu were generated through GeneArt™ Site-Directed Mutagenesis System (Invitrogen, Carlsbad, CA, USA). Then, they were co-transfected with pRL-TK vectors (Shanghai Yihui Biological Technology Co., Ltd., Shanghai, China) and miR-143 mimic or miR-control into MGC-803 and GES-1 cells. Luciferase activities were determined after 48h.

### Invasion assay

Transwell invasion assay was performed as described in the earlier study 16. The migrated cells in random three visual fields were photographed and counted under a microscope (Olympus, Tokyo, Japan).

### Statistical Analysis

All experiments were performed in triplicate unless specified. Results were represented as the Mean ± SEM. Statistical analysis was performed using unpaired Student's *t*-test or *F* test. The overall survival rate of the patients was measured by the Kaplan-Meier method, and the survival rate between the groups was compared by a log-rank test. *P*<0.05 was considered significant.

## Results

### hsa_circRNA_101996 is upregulated in GC tissues

**Figure 1:**
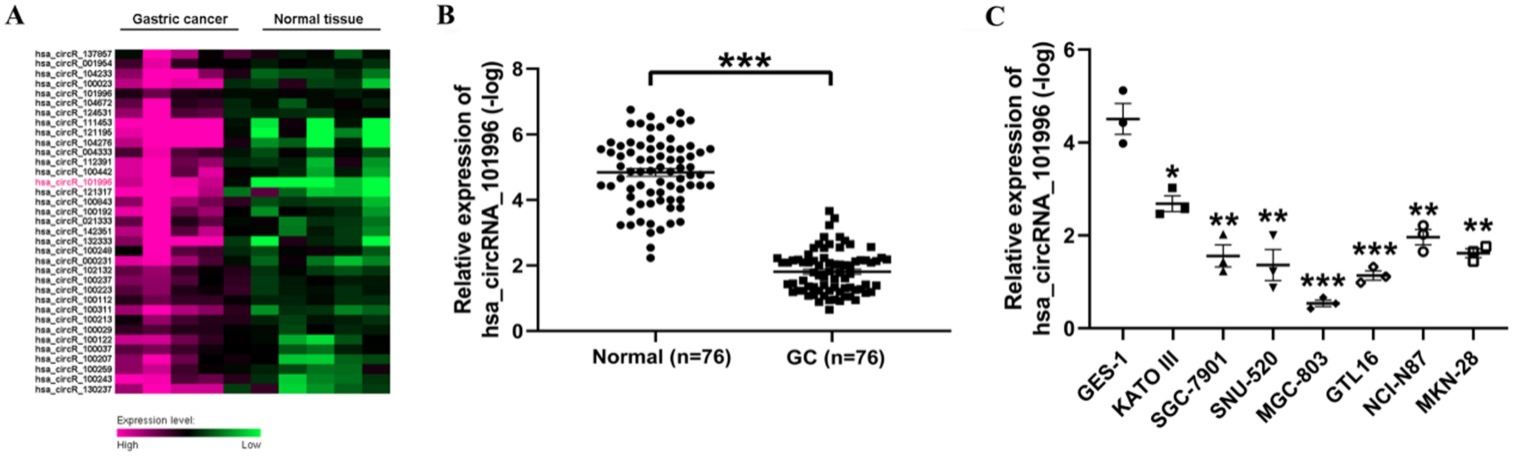
** hsa_circRNA_101996 is upregulated in gastric cancer tissues. (A)** The differentially expressed circRNA in gastric cancer and normal tissues through data set GSE3234. **(B)** hsa_circRNA_101996 levels in gastric cancer tissues and the normal tissues. **(C)** hsa_circRNA_101996 levels in gastric cancer cell lines. *, *P*<0.05; **, *P*<0.01; ***, *P*<0.001.

First, we used the data set GSE3234 to analyze the differentially expressed circRNA in GS and normal tissues. It was found that hsa_circRNA_101996 is highly expressed in GC tissues (Figure 1A). Then, we analyzed the hsa_circRNA_101996 expression level in GC and the adjacent tissue. It showed that the level of hsa_circRNA_101996 was significantly increased in GC tissues compared to that in the normal tissues (Figure 1B). The expression pattern of hsa_circRNA_101996 was also analyzed in GC cell lines and human gastric mucosal epithelium cell line GES-1. Compared to GES-1, hsa_circRNA_101996 was markedly upregulated in GC cell lines, among which, the up-regulation of hsa_circRNA_101996 was most significant in MGC-803 (Figure 1C).

### hsa_circRNA_101996 enhances cell invasion and is related to metastasis and poor prognosis of GC

**Figure 2:**
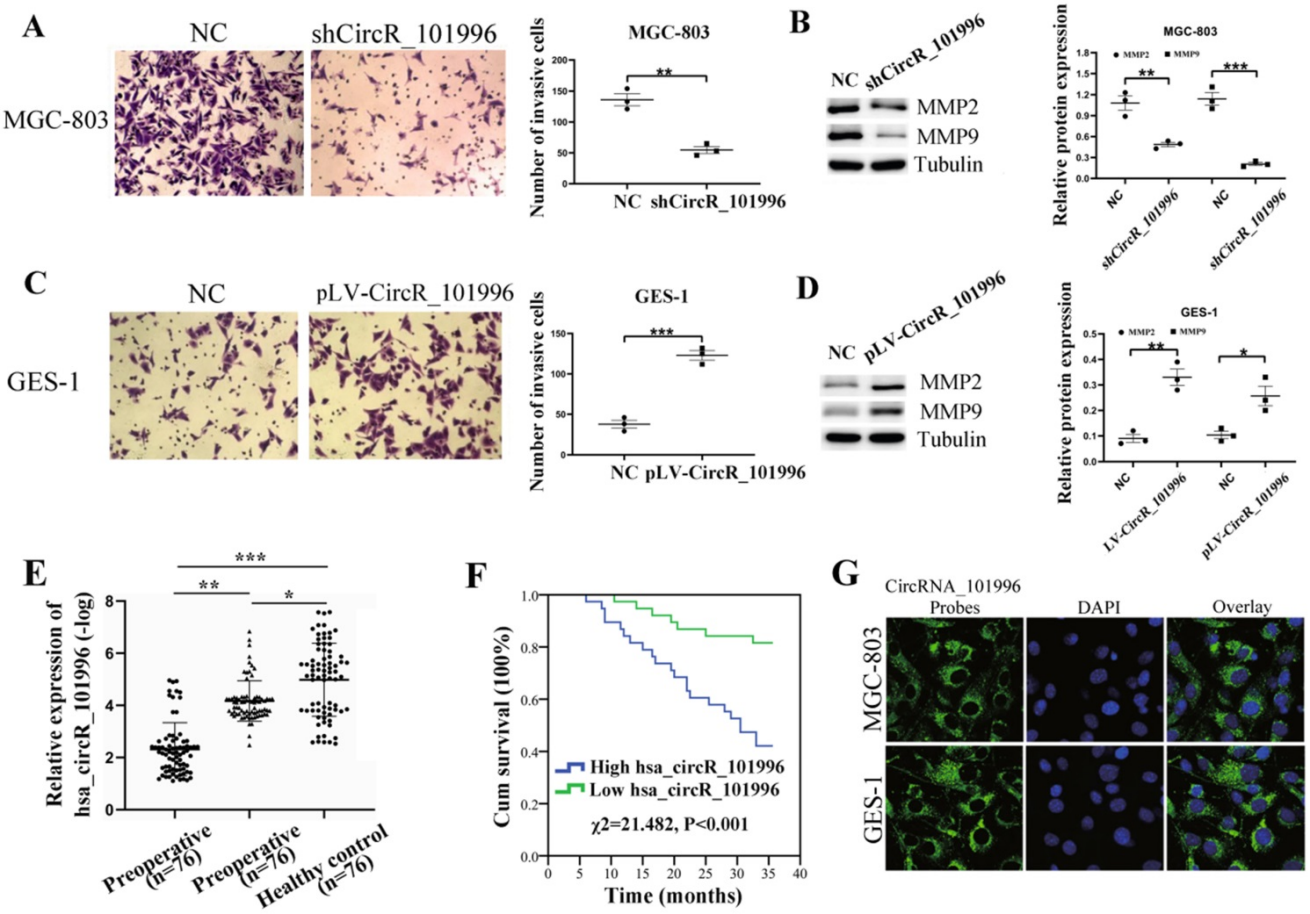
** hsa_circRNA_101996 enhances cell invasion and is related to the metastasis and poor prognosis of gastric cancer. (A)** Comparing to the NC group, shCircR_101996 significantly inhibited the invasion ability of MGC-803. **(B)** Comparing to the NC group, shCircR_101996 significantly down-regulated the expression of MMP2/MMP9. **(C)** Comparing to the NC group, overexpression of hsa_circRNA_101996 significantly enhanced cell invasion of GES-1. **(D)** Comparing to the NC group, overexpression of hsa_circRNA_101996 significantly enhanced the expressions of MMP2/MMP9. **(E)** The postoperative plasma hsa_circRNA_101996 level of gastric cancer patients was significantly lower than that of preoperative patients, but it was still significantly higher than that of healthy controls. **(F)** Compared to the low hsa_circRNA_101996 expression group, the three-year survival rate of patients in the high hsa_circRNA_101996 expression group was significantly lower (χ^2^=21.482, *P*<0.001). **(G)** FISH experiments with probes targeting hsa_circRNA_101996 were performed to validate the subcellular localization of hsa_circRNA_101996 in MGC-803 and GES-1 cells, the cytoplasm was stained with probes targeting hsa_circRNA_101996 (green stain), and the nuclei were stained with DAPI (blue stain). *, *P*<0.05; **, *P*<0.01; ***, *P*<0.001.

We analyzed the correlation between hsa_circRNA_101996 and the clinical characteristics of patients. The results showed that hsa_circRNA_101996 expression in GC tissues is significantly correlated with tumor size, lymphatic metastasis, and distant metastasis (Table 1). MGC-803 and GES-1 cell lines were then used to investigate the function of hsa_circRNA_101996 in GC. Results revealed that compared to the NC group, shCircR_101996 significantly inhibited the invasion ability of MGC-803 (Figure 2A), and down-regulated the expressions of MMP2/MMP9 (Figure 2B), while overexpression of hsa_circRNA_101996 in GES-1 significantly enhanced cell invasion (Figure 2C), as well as the expressions of MMP2/MMP9 (Figure 2D). These results indicate that hsa_circRNA_101996 promotes GC development by inducing MMP2/MMP9 expression.

Then, we used qPCR to detect the plasma hsa_circRNA_101996 expression levels of GC patients before and after surgery and healthy controls. We found that the hsa_circRNA_101996 level in postoperative plasma of GC patients was significantly lower than that in preoperative patients, but it was still significantly higher than that in healthy controls (Figure 2E). According to the median of the postoperative plasma hsa_circRNA_101996 expression level, 76 patients were divided into two groups. Compared with the low hsa_circRNA_101996 expression group, the three-year survival rate of patients in the high hsa_circRNA_101996 expression group was significantly lower (χ^2^=21.482, *P*<0.001, Figure 2F). The FISH and nuclear/cytoplasmic RNA-separation experiments showed that hsa_circRNA_101996 was mainly localized in the cytoplasm in MGC-803 and GES-1 cells (Figure 2G), indicating that hsa_circRNA_101996 might exert functions through the ceRNA network.

### hsa_circRNA_101996 sponges miR-143 to regulate TET2 expression

**Figure 3:**
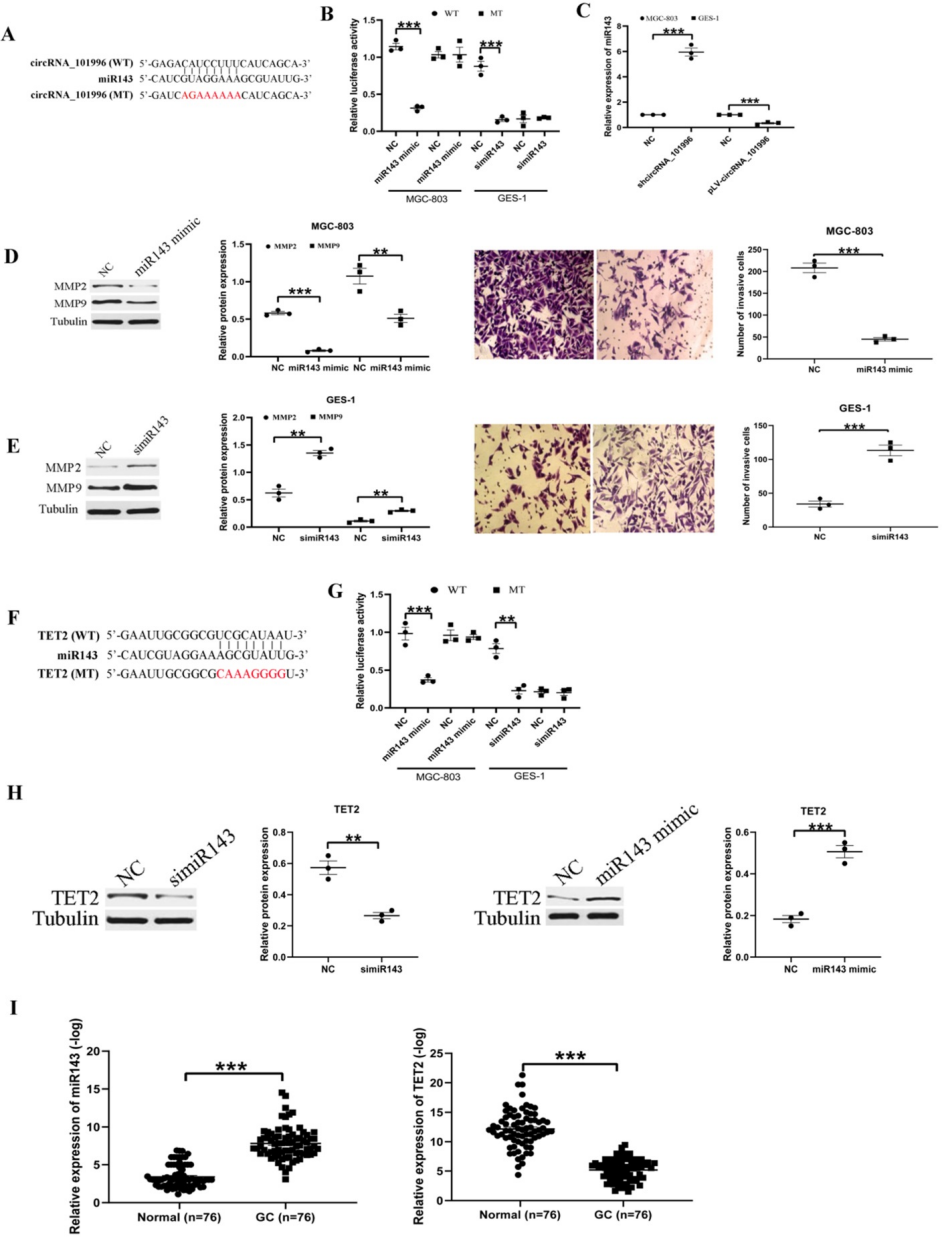
** hsa_circRNA_101996 sponges miR-143 to regulate TET2 expression. (A)** hsa_circRNA_101996 could bind to miR-143. **(B)** Overexpression of miR-143 significantly weakened the luciferase activity of wide‐type hsa_circRNA_101996, while mutation of the binding site blocked the inhibitory effect. **(C)** Knockdown of hsa_circRNA_101996 in MGC-803 increased miR-143 level, while its overexpression in GES-1 led to the decrease of miR-143. **(D-E)** Overexpression of miR-143 in MGC-803 significantly inhibited the expressions of MMP2/MMP9 and the invasion ability of cells, while knockdown of miR-143 in GES-1 led to the enhanced cell invasion and the increased MMP2/MMP9 expression. **(F)** miR-143 could bind to the 3'-UTR regions of TET2 mRNA. **(G)** Overexpression of miR-143 significantly weakened the luciferase activity of wide-type TET2, while mutation of the binding site blocked the inhibitory effect. **(H)** Overexpression of miR-143 in MGC-803 significantly inhibited the expressions of TET2, while knockdown of miR-143 in GES-1 led to the increased TET2 expression. **(I)** miR-143 level decreased markedly in gastric cancer tissue compared to that in the adjacent tissue, while the change of TET2 expression was contrary to miR-143. **, *P*<0.01; ***, *P*<0.001.

To explore the mechanism of hsa_circRNA_101996 in GC development, we analyzed its possible miR partner. The results showed that hsa_circRNA_101996 could bind to miR-143 (Figure 3A), and this was confirmed by luciferase assays: overexpression of miR-143 significantly weakened the luciferase activity of wide‐type hsa_circRNA_101996, while mutation of the binding site blocked the inhibitory effect (Figure 3B). Besides, knockdown of hsa_circRNA_101996 in MGC-803 increased miR-143 level, while overexpression of hsa_circRNA_101996 in GES-1 led to the decrease of miR-143 (Figure 3C). Overexpression of miR-143 in MGC-803 significantly inhibited the expressions of MMP2/MMP9 and the invasion ability of cells, while knockdown of miR-143 in GES-1 led to the enhanced cell invasion and the increased MMP2/MMP9 expression (Figure 3D, E). We next analyzed the target mRNA of miR-143 using bioinformatics analysis. The results showed that miR-143 could bind to the 3'-UTR regions of TET2 mRNA (Figure 3F). The relationship between miR-143 and TET2 was confirmed by the luciferase assays: overexpression of miR-143 significantly weakened the luciferase activity of wide-type TET2, while mutation of the binding site blocked the inhibitory effect (Figure 3G). Besides, we observed that the expression of TET2 was regulated by hsa_circRNA_101996/miR-143. Overexpression of miR-143 in MGC-803 significantly inhibited the expressions of TET2, while knockdown of the miR-143 in GES-1 increased the expression of TET2 (Figure 3H). We also analyzed the expression of miR-143 and TET2 in human GC tissues and the adjacent tissues. The results showed that the level of miR-143 decreased markedly in GC tissues compared to that in the adjacent tissues, while the change of TET2 expression was contrary to miR-143 (Figure 3I). Plasma hsa_circRNA_101996 and miR-143 levels in GC patients are significantly negatively correlated (*r*=-0.542, *P*<0.001).

### TET2 regulates MMP2/MMP9 expression through the DNA demethylation pathway

**Figure 4:**
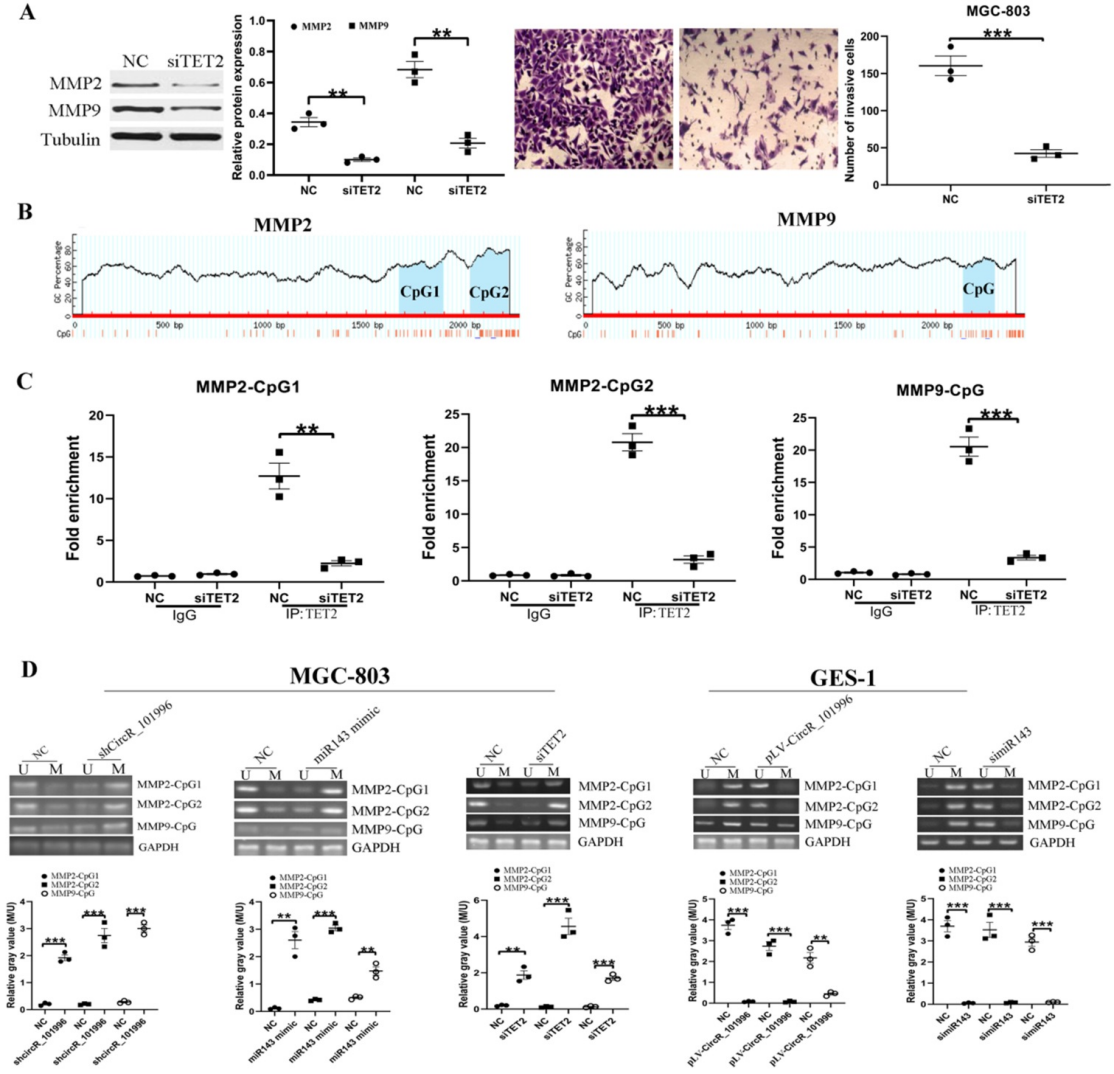
** TET2 regulates MMP2/MMP9 expression through the epigenetic pathway. (A)** MGC-803 showed lower MMP2/MMP9 expression and decreased invasive ability after TET2 knockdown. **(B-C)** ChIP assay showed that TET2 could bind to the CpG islands in the promoter and Exon 1 of the MMP2/MMP9 gene directly. **(D)** Overexpression of hsa_circRNA_101996 and knockdown of miR-143 could both lead to the hypermethylation of CpG islands. **, *P*<0.01; ***, *P*<0.001.

To clarify the relationship between TET2 and MMP2/MMP9, MGC-803 was transfected with siTET2 to knock down TET2 expression. Compared to the NC group, the expression of MMP2/MMP9 and the cell invasive ability were decreased in the siTET2 group (Figure 4A). ChIP assay showed that TET2 could bind to the CpG islands in the promoter and Exon 1 of the MMP2/MMP9 gene directly (Figure 4B, C). We next analyzed the DNA methylation levels of CpG islands in the MMP2/MMP9 promoter and Exon 1. As the results showed, in MGC-803 cells, knockdown of hsa_circRNA_101996, overexpression of miR-143, and knockdown of TET2 could all enhanced the methylation of CpG islands, while in GES-1 cells, overexpression of hsa_circRNA_101996 and knockdown of miR-143 could both lead to the hypermethylation of CpG islands (Figure 4D).

### hsa_circRNA_101996 inhibit tumor growth by miR-143/TET2 axis *in vivo*

**Figure 5:**
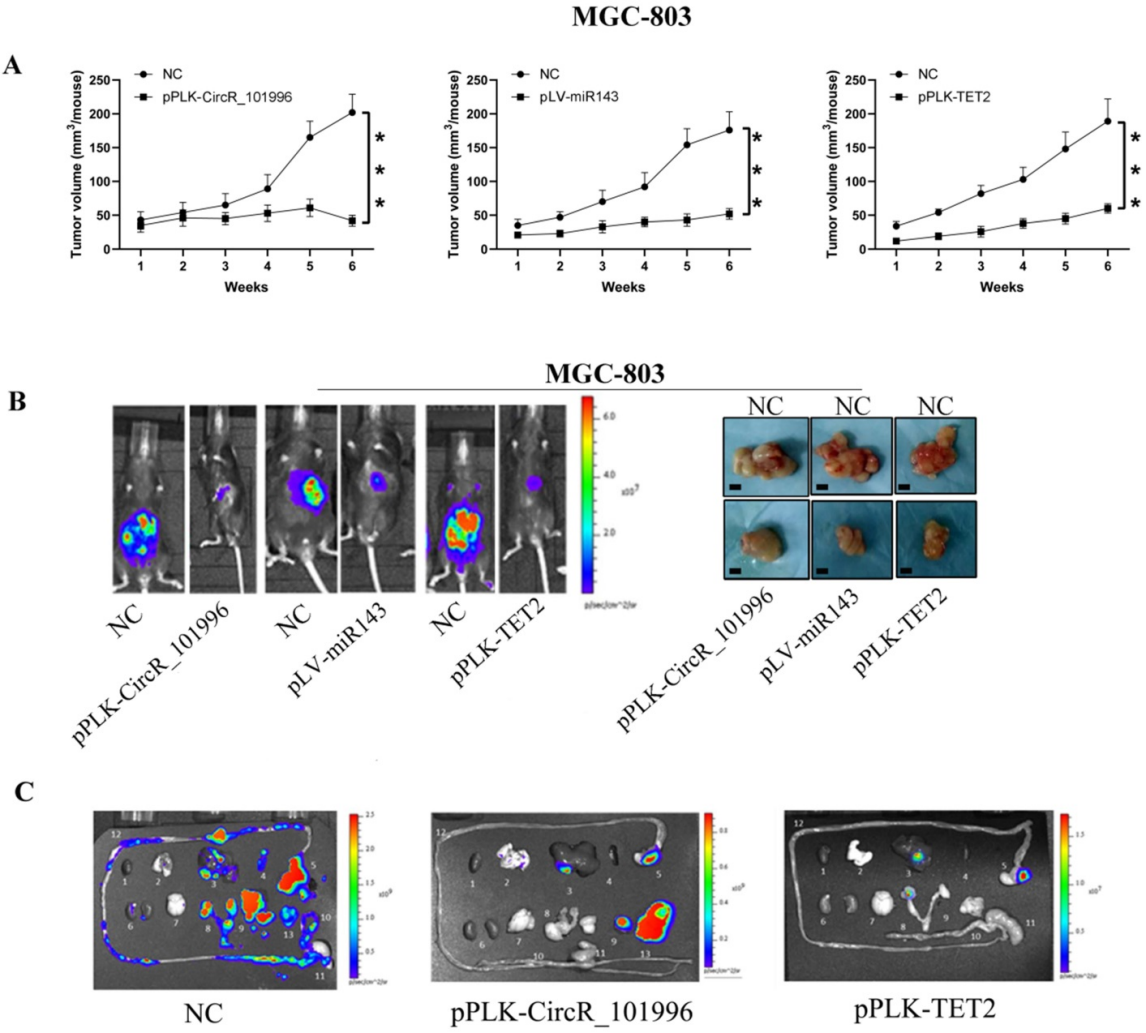
** Suppressing hsa_circRNA_101996/TET2 or overexpressing miR-143 inhibits tumor growth. (A-B)** pPLK-CircR_101996, pPLK-TET2, and pLV-miR-143 all markedly reduced the tumor size of gastric cancer. The scale bar is 5 mm. **(C)** The pPLK-CircR_101996 and pPLK-TET2 could both inhibit abdominal metastasis. ***, *P*<0.001.

To verify the therapeutic value of hsa_circRNA_101996/miR-143/TET2, MGC-803 with stably knockdown of hsa_circRNA_101996/TET2 or stably expressing miR-143 were injected into the serious side of the stomach of mice. Compared to MGC-803, another GC cell line KATO III expressed hsa_circRNA_101996 at a lower level. KATO III stably overexpressing hsa_circRNA_101996, or knockdown of miR-143 was also used for the modeling. The results showed that pPLK-CircR_101996, pPLK-TET2, and pLV-miR-143 all markedly reduced the tumor size of GC (Figure 5A, B), while the pPLK-CircR_101996 and pPLK-TET2 could both inhibit abdominal metastasis (Figure 5C). These results suggest that hsa_circRNA_101996/TET2/miR-143 could be the therapeutic target of GC by inhibiting the invasion of cancer cells.

## Discussion

Growing studies have revealed that circRNAs have the function of gene regulation 17. Increasing evidence has suggested that many CircRNAs, are closely related to the process of cancer development 2. CircRNA functions as a miR sponge to reduced miR expression and function further to indirectly regulate the expression of target genes 18. hsa_circRNA_101996 is associated with cervical cancer 19,20. So far, the association between GC and hsa_circRNA_101996 has not been studied much. Therefore, we initially tested whether hsa_circRNA_101996 was dysregulated in GC, clarified its effect on GC cell proliferation *in vitro*, and clarified the underlying mechanisms.

Our results revealed that hsa_circRNA_101996 expression was significantly upregulated in GC tissues compared to that in the adjacent tissue, and its level in cancer tissues is correlated with tumor size, lymphatic metastasis, and distant metastasis. Suppression of hsa_circRNA_101996 inhibited the migration and invasion of GC cells as well as tumor growth *in vivo*. In contrast, overexpression of hsa_circRNA_101996 promoted the migration and invasion of GES-1cells.

It has been reported that hsa_circRNA_101996 was upregulated in cervical cancer and played a key role in disease progression. For instance, Song T et al. 19 suggested that higher hsa_circRNA_101996 expression would lead to poor survival of cervical cancer patients after surgery, and hsa_circRNA_101996 could promote SiHa and CaSki cell proliferation and invasion by sponging miR‐8075 to promote TPX2 expression. One circRNA might simultaneously sponge several miRs. Moreover, they uncovered that miR-1236-3p can also be targeted by hsa_circRNA_101996 in cervical cancer. hsa_circRNA_101996 up-regulated TRIM37 expression by suppressed miR-1236-3p to promoted cervical cancer development 20. In our study, we found that knockdown hsa_circRNA_101996 significantly inhibited the invasion ability of MGC-803 and decreased the expression of MMP2/MMP9. On the contrary, overexpress hsa_circRNA_101996 significantly promoted the invasion ability of GES-1 and increased the expression of MMP2/MMP9. It suggested that hsa_circRNA_101996 may serve as an oncogene in GC.

We next explored the molecular mechanisms underlying the oncogenic action of hsa_circRNA_101996 in GC. As we know, circRNAs can perform biological functions by acting as molecular sponges to inactivate miRs. MiR-143 has been reported to be downregulated in esophageal cancer 21, bladder cancer 22, pancreatic ductal adenocarcinoma 23, and lung cancer 24, indicating that miR-143 may exert tumor-suppressive effects in these cancers. Our results showed that hsa_circRNA_101996 could bind to miR-143. Moreover, knockdown of hsa_circRNA_101996 in MGC-803 increased the miR-143 level, while its overexpression in GES-1 led to the decrease of miR-143. Our data showed that the expression of miR-143 was accordingly decreased in GC tissues compared to the adjacent tissues.

Furthermore, another important finding of our study was that TET2 was verified as a target of miR-143, and TET2 expression was negatively regulated by miR-143. Besides, suppressing hsa_circRNA_101996/TET2 or overexpressing miR-143 inhibits tumor growth *in vivo*. TET2 was reported more associated with gene bodies and enhancers than with CpG-rich promoters 25. MMP-2 and MMP-9 are two key enzymes in the process of cancer cell invasion and metastasis. The activation of the two can form type IV collagenase, degrade the extracellular matrix, destroy the complete basement membrane, promote the infiltration of surrounding tissues by cancer cells, invade blood vessels and lymphatic vessels, and metastasize 26,27. In this study, we found that in MGC-803 cells, knockdown of hsa_circRNA_101996, overexpression of miR-143, and knockdown of TET2 could all enhanced the methylation of CpG islands, while in GES-1 cells, overexpression of hsa_circRNA_101996 and knockdown of miR-143 could both lead to the hypermethylation of CpG islands.

In summary, our data revealed that hsa_circRNA_101996 over-expression promoted the cell migration and invasion of GC cells, while hsa_circRNA_101996 knockdown exerted opposite effects. Moreover, hsa_circRNA_101996 negatively modulated miR-143 and decreased its expression. The axis of hsa_circRNA_101996/miR-143 exerted an important function by regulating TET2 expression in GC. Besides, the effect of hsa_circRNA_101996/miR-143/TTE2 on GC growth was confirmed in a mouse xenograft model. The axis of hsa_circRNA_101996/miR-143/TET2 has the potential to be investigated as the therapeutic target for the treatment of GC.

## Figures and Tables

**Table 1:** The correlation between hsa_circRNA_101996/miR143/TET2 level (-log) in gastric cancer tissue and the clinical characteristics of patients

Clinical characteristics	hsa_circRNA_101996			miR143			TET2		
	≤ median	> median	*P*	≤ median	> median	*P*	≤ median	> median	*P*
**Gender**			0.82			0.25			0.11
Male	18	19		21	16		15	22	
Female	20	19		17	22		23	16	
**Age**			0.49			0.11			0.82
>50	23	20		25	18		22	21	
<50	15	18		13	20		16	17	
**History of Helicobacter pylori infection**			0.53			0.21			0.12
Yes	31	33		34	30		29	35	
No	7	5		4	8		9	3	
**Family history of gastric cancer**			0.50			1.00			0.18
Yes	6	4		5	5		7	3	
No	32	34		33	33		31	35	
**Body Mass Index (kg/m^2^)**			0.30			0.60			0.12
>24	26	30		27	29		25	31	
≤24	12	8		11	9		13	7	
**Tumor size (cm)**			<0.01			<0.01			<0.01
>3	12	30		35	7		10	32	
≤3	26	8		3	31		28	6	
**TNM stage**			<0.01			<0.01			<0.01
1-2	11	35		31	15		13	33	
3-4	27	3		7	23		25	5	

## Abbreviations

circRNAs: circular RNAs
ChIP: Chromatin immunoprecipitation
GC: gastric cancer
MMPs: matrix metalloproteinases
miR: microRNA
MSP: methylation-specific polymerase chain reaction
Mu: mutant type
qRT-PCR: quantitative reverse transcription-polymerase chain reaction
TET: ten-eleven translocation
WT: wide type
