# Circular RNA Hsa_circRNA_101996 promotes the development of Gastric Cancer via Upregulating Matrix Metalloproteinases-2/Matrix Metalloproteinases-9 through MicroRNA-143/Ten-eleven translocation-2 Pathway: Erratum

**DOI:** 10.7150/jca.96046

**Published:** 2024-03-22

**Authors:** Feng Huang, Jiajia Jiang, Yongliang Yao, Shiyue Hu, He Wang, Ma Zhu, Liya Yu, Qingqian Liu, Haoyuan Jia, Wenrong Xu

**Affiliations:** 1 Jiangsu Key Laboratory of Medical Science and Laboratory Medicine, School of Medicine, Jiangsu University, 301 Xuefu Road, Zhenjiang, Jiangsu 212013, China.; 2 Department of Clinical Laboratory, The First People's Hospital of Kunshan Affiliated with Jiangsu University, Kunshan, 215300, China.; 3 Aoyang Institute of Cancer, Jiangsu University, 279 Jingang Road, Suzhou, 215600, Jiangsu, China.; 4 Cancer Research Institute of Wuhan, The Central Hospital of Wuhan, Tongji Medical College, Huazhong University of Science and Technology, Wuhan 430014, China.; 5 Department of Clinical Laboratory, Wuxi People's Hospital Affiliated to Nanjing Medical University, Wuxi, China.

We regret that the original version of our paper unfortunately contained incorrect representative images. The wrong images were placed in Figures 2A-D, 2G, 3D, 3E, 3H, 4A, 5B and 5C when choosing representative images from very large amount of data. The correct version of the Figures 2A-D, 2G, 3D, 3E, 3H, 4A, 5B and 5C appears below. The authors confirm that the corrections made in this erratum do not affect the original conclusions. All the authors of the paper have agreed to this correction. The authors apologize for any inconvenience that the errors may have caused.

## Figures and Tables

**Figure 2:**
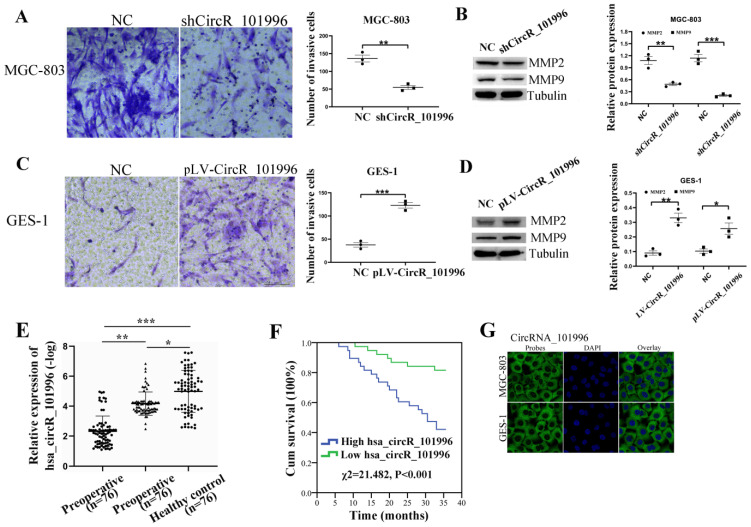
hsa_circRNA_101996 enhances cell invasion and is related to the metastasis and poor prognosis of gastric cancer. (A) Comparing to the NC group, shCircR_101996 significantly inhibited the invasion ability of MGC-803. (B) Comparing to the NC group, shCircR_101996 significantly down-regulated the expression of MMP2/MMP9. (C) Comparing to the NC group, overexpression of hsa_circRNA_101996 significantly enhanced cell invasion of GES-1. (D) Comparing to the NC group, overexpression of hsa_circRNA_101996 significantly enhanced the expressions of MMP2/MMP9. (E) The postoperative plasma hsa_circRNA_101996 level of gastric cancer patients was significantly lower than that of preoperative patients, but it was still significantly higher than that of healthy controls. (F) Compared to the low hsa_circRNA_101996 expression group, the three-year survival rate of patients in the high hsa_circRNA_101996 expression group was significantly lower (*χ^2^*=21.482, *P*<0.001). *, *P*<0.05; **, *P*<0.01; ***, *P*<0.001.

**Figure 3:**
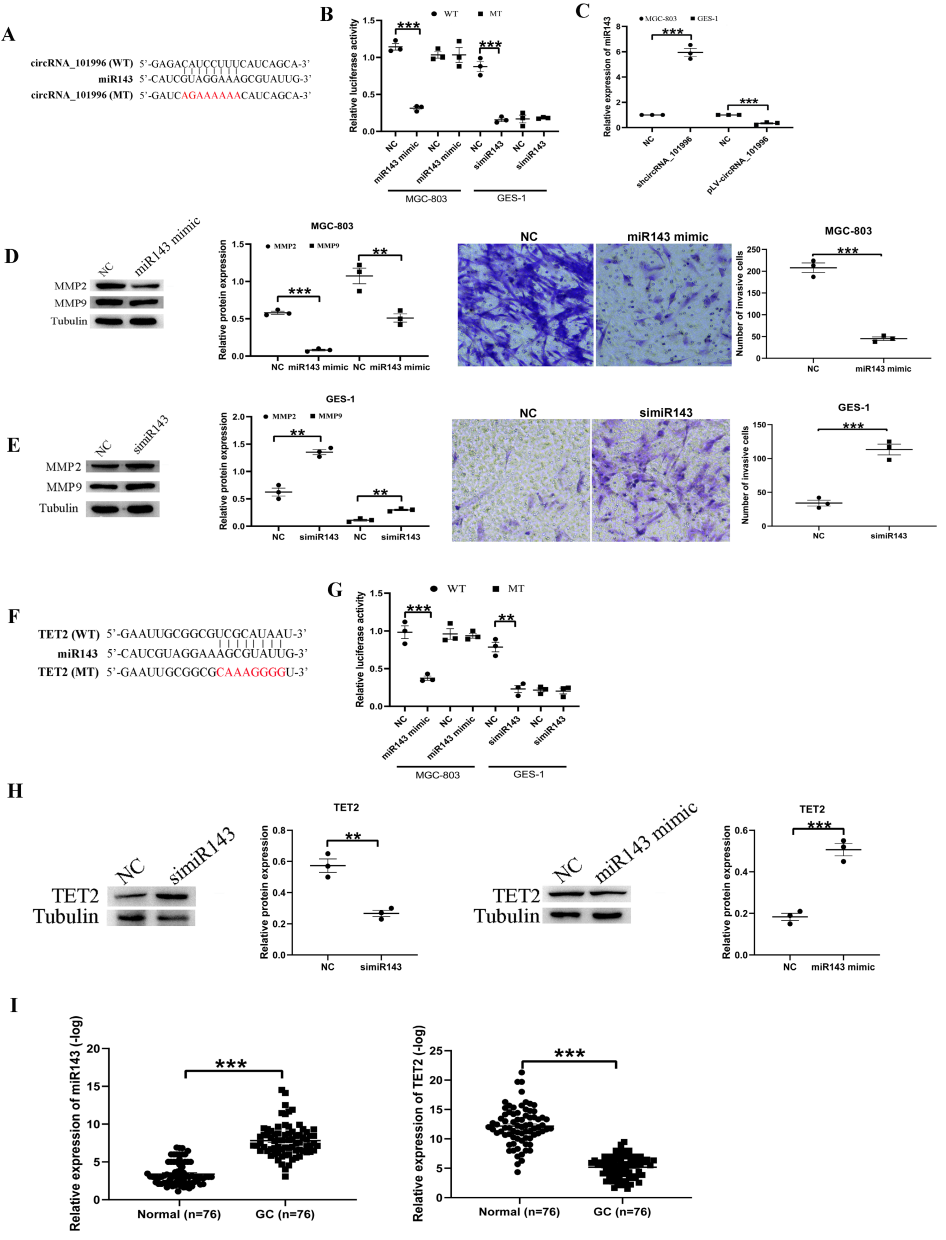
hsa_circRNA_101996 sponges miR-143 to regulate TET2 expression. (A) hsa_circRNA_101996 could bind to miR-143. (B) Overexpression of miR-143 significantly weakened the luciferase activity of wide‐type hsa_circRNA_101996, while mutation of the binding site blocked the inhibitory effect. (C) Knockdown of hsa_circRNA_101996 in MGC-803 increased miR-143 level, while its overexpression in GES-1 led to the decrease of miR-143. (D-E) Overexpression of miR-143 in MGC-803 significantly inhibited the expressions of MMP2/MMP9 and the invasion ability of cells, while knockdown of miR-143 in GES-1 led to the enhanced cell invasion and the increased MMP2/MMP9 expression. (F) miR-143 could bind to the 3'-UTR regions of TET2 mRNA. (G) Overexpression of miR-143 significantly weakened the luciferase activity of wide-type TET2, while mutation of the binding site blocked the inhibitory effect. (H) Overexpression of miR-143 in MGC-803 significantly inhibited the expressions of TET2, while knockdown of miR-143 in GES-1 led to the increased TET2 expression. (I) miR-143 level decreased markedly in gastric cancer tissue compared to that in the adjacent tissue, while the change of TET2 expression was contrary to miR-143. **, *P*<0.01; ***, *P*<0.001.

**Figure 4:**
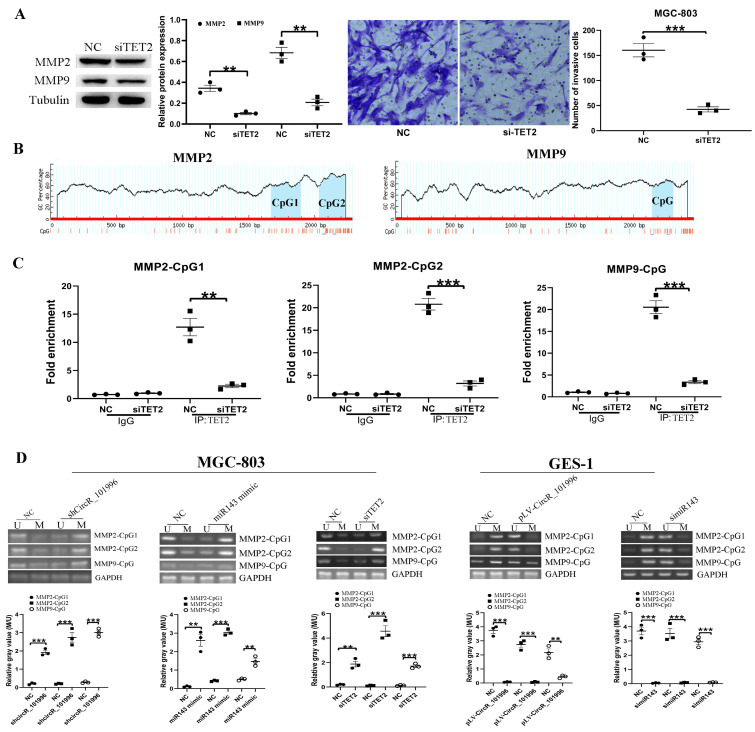
TET2 regulates MMP2/MMP9 expression through the epigenetic pathway. (A) MGC-803 showed lower MMP2/MMP9 expression and decreased invasive ability after TET2 knockdown. (B-C) ChIP assay showed that TET2 could bind to the CpG islands in the promoter and Exon 1 of the MMP2/MMP9 gene directly. (D) Overexpression of hsa_circRNA_101996 and knockdown of miR-143 could both lead to the hypermethylation of CpG islands. **, *P*<0.01; ***, *P*<0.001.

**Figure 5:**
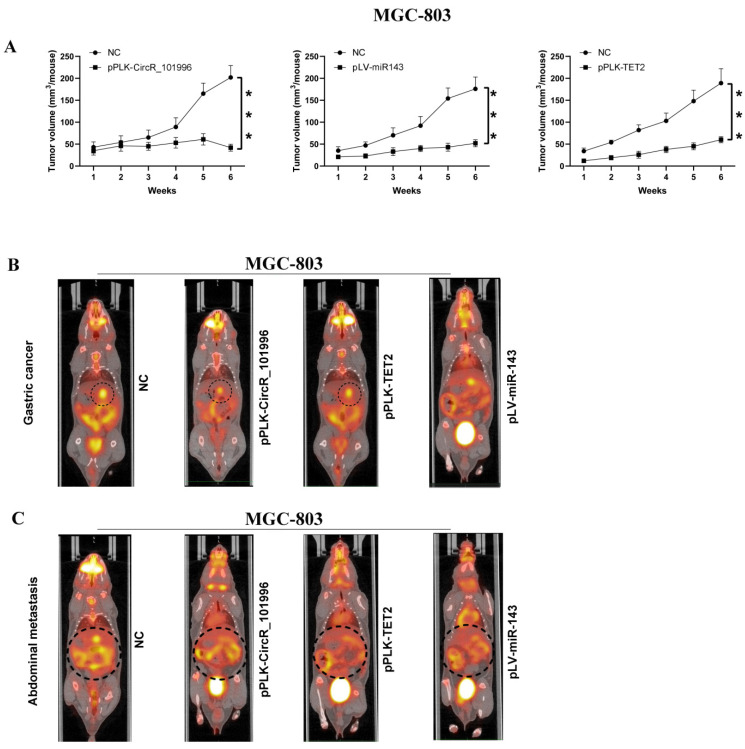
Suppressing hsa_circRNA_101996/TET2 or overexpressing miR-143 inhibits tumor growth. (A-B) pPLK-CircR_101996, pPLK-TET2, and pLV-miR-143 all markedly reduced the tumor size of gastric cancer. The scale bar is 5 mm. (C) The pPLK-CircR_101996 and pPLK-TET2 could both inhibit abdominal metastasis. ***, *P*<0.001.

